# Biodegradable HPMC–chitosan film for moisture retention and quality preservation in fresh‐cut mango

**DOI:** 10.1002/jsfa.70393

**Published:** 2025-12-18

**Authors:** Angelucia Gonçalves Parente, Geraldo Vieira de Lima Júnior, Ana Caroliny de Souza, Fernanda Silva Ferreira, Pedro Vitor Moura Rocha, Mariana Paola Cabrera, Sérgio Tonetto de Freitas, David Fernando de Morais Neri

**Affiliations:** ^1^ Institute of Materials Science, Universidade Federal do Vale do São Francisco Juazeiro Brazil; ^2^ Department of Fundamental Chemistry Universidade Federal de Pernambuco Recife Brazil; ^3^ Postharvest Physiology and Technology Laboratory, Brazilian Agricultural Research Corporation Petrolina Brazil

**Keywords:** biodegradable packaging, climacteric fruit, food preservation, minimally processed, polysaccharide‐based films

## Abstract

**BACKGROUND:**

Replacing conventional plastics with biodegradable solutions that control moisture loss in fresh‐cut produce is a strategic need. This study developed hydroxypropylmethylcellulose (HPMC) and chitosan (CS) films plasticised with glycerol (Gly) and evaluated their use as sealing lids for fresh‐cut mango.

**RESULTS:**

The selected monolayer formulation showed high optical clarity with limited transmittance at 560 nm, reduced water solubility and water‐vapour permeability suited to moisture management. Attenuated total reflection Fourier transform infrared analysis indicated HPMC–CS compatibility and scanning electron microscopy imaging showed a continuous surface. Applied at 9 °C for nine days, the HPMC/CS+Gly film reduced weight loss by about fivefold relative to unpackaged fruit and approached the performance of commercial poly(vinyl chloride) in limiting dehydration, while maintaining fruit colour (Lab*) and firmness during storage. Package headspace measurements were consistent with a performance profile focused on moisture control.

**CONCLUSION:**

The HPMC/CS+Gly film is a promising biodegradable option for mitigating dehydration and preserving quality in fresh‐cut mango, and it provides a robust platform for future optimisation of gas‐barrier properties and, where appropriate, incorporation of active functionalities. © 2025 The Author(s). *Journal of the Science of Food and Agriculture* published by John Wiley & Sons Ltd on behalf of Society of Chemical Industry.

## INTRODUCTION

As consumer demand for convenient, safe and high‐quality foods continues to rise, food packaging has become a strategic component in modern food systems, contributing to containment, protection, communication and product integrity.^1^ Beyond these essential functions, packaging plays a pivotal role in maintaining the microbiological and physicochemical quality of foods, directly influencing shelf life and minimising postharvest losses.^1^, ^2^ These aspects are particularly critical for fresh‐cut fruits and vegetables, which have gained increasing popularity due to their practicality and nutritional value.^3^, ^4^

Nevertheless, maintaining the quality of fresh‐cut products remains a complex challenge due to their high metabolic activity, structural fragility and susceptibility to microbial spoilage.^4^, ^5^ These characteristics accelerate moisture loss, oxidative browning and microbial growth, ultimately leading to a shortened shelf life and increased risk of consumer rejection. Even under refrigeration and combined with physical or chemical preservation techniques, the retention of key attributes such as firmness, colour, juiciness and flavour is often compromised.^3^, ^6^, ^7^, ^8^

Among preservation strategies, approaches that modulate the package atmosphere have been widely investigated to slow respiration and metabolic processes and, in some cases, to delay tissue senescence and preserve quality.^9^, ^10^, ^11^, ^12^, ^13^, ^14^, ^15^, ^16^ Such approaches have shown success across a range of fresh‐cut produce,^17^, ^18^, ^19^ while polymeric films remain central because they govern gas permeability, moisture transfer and light exposure, and provide mechanical protection.^17^, ^18^, ^19^, ^20^, ^21^ At the same time, environmental concerns regarding conventional synthetic plastics have spurred efforts to develop biodegradable alternatives from renewable sources.^22^, ^23^ In this context, biobased polymers have emerged as sustainable options that can offer suitable barrier properties and reduce the environmental footprint of packaging materials.^20^, ^24^, ^25^, ^26^

Among these alternatives, hydroxypropylmethylcellulose (HPMC) and chitosan (CS) stand out due to their excellent film‐forming capabilities, biodegradability and safety for food applications.^27^, ^28^ HPMC films exhibit high transparency, flexibility, low oxygen permeability and resistance to fats,^29^ although their hydrophilic nature and relatively high production costs may limit effectiveness in high‐moisture systems.^30^, ^31^ CS, the second most abundant natural polysaccharide after cellulose and derived from chitin,^32^, ^33^ may display inherent antimicrobial activity associated with its cationic nature and also shows good biocompatibility and gas‐barrier potential,^34^, ^35^ though its low mechanical strength can restrict standalone application.^36^ Blending HPMC and CS has therefore proven effective, enabling composite films with enhanced mechanical strength, cohesive structure and improved functional performance.^34^ Incorporating glycerol (Gly) as a plasticiser reduces intermolecular forces, increasing flexibility, preventing shrinkage and facilitating handling and storage.^37^, ^39^

Given the high perishability and economic relevance of mango in tropical supply chains, improving moisture management for fresh‐cut mango is particularly pertinent. Therefore, the study reported here aimed to develop and characterise a biodegradable HPMC–CS film plasticised with Gly and to evaluate its performance in limiting moisture loss and preserving product quality (weight loss, colour and firmness) in fresh‐cut mango under refrigerated storage, using commercial poly(vinyl chloride) (PVC) film and an unpackaged control as benchmarks.

## MATERIALS AND METHODS

### Materials

Commercial CS (from shrimp shells, minimum deacetylation degree 75%, Sigma‐Aldrich®), HPMC (viscosity 40–60 cP in 2% aqueous solution at 20 °C, Sigma‐Aldrich®), Gly (purity > 99.5%, Sigma‐Aldrich®) and glacial acetic acid (analytical grade) were used in this study.

### Preparation of film‐forming solutions and monolayer films

HPMC and CS solutions were prepared separately. HPMC was dispersed in distilled water and stirred using a magnetic stirrer in a water bath at 75 °C for 1 h. After complete dissolution, the solution was cooled to room temperature (25 °C) and manually stirred for 20 min, followed by magnetic stirring for an additional 30 min to ensure homogeneity and transparency. CS was dissolved in 1.0% (v/v) acetic acid and stirred at 50 °C for 1 h. The resulting solution was vacuum‐filtered to remove insoluble residues.

Equal volumes of the HPMC and CS solutions were combined and magnetically stirred at room temperature for 10 min. The concentrations presented in Table 1 refer to the individual polymer solutions prior to mixing; therefore, the 1:1 blending step resulted in final concentrations equal to half of the initial values, which was intentionally accounted for in the formulation design. Gly was then added at the concentrations established by the factorial experimental design (Table 1), and the mixture was stirred for an additional 20 min.

**Table 1:** Coded and actual values of the independent variables in the 2^3^ factorial design for HPMC–CS–Gly film formulations

Standard run^a^	Coded/[HPMC] (%)	Coded/[CS] (%)	Coded/[Gly] (%)
1	−1 (1.00)	−1 (0.50)	−1 (0.50)
2	1 (3.00)	−1 (0.50)	−1 (0.50)
3	−1 (1.00)	1 (1.00)	−1 (0.50)
4	1 (3.00)	1 (1.00)	−1 (0.50)
5	−1 (1.00)	−1 (0.50)	1 (2.00)
6	1 (3.00)	−1 (0.50)	1 (2.00)
7	−1 (1.00)	1 (1.00)	1 (2.00)
8	1 (3.00)	1 (1.00)	1 (2.00)
9 (C)	0 (2.00)	0 (0.75)	0 (1.00)
10 (C)	0 (2.00)	0 (0.75)	0 (1.00)
11 (C)	0 (2.00)	0 (0.75)	0 (1.00)
12 (C)	0 (2.00)	0 (0.75)	0 (1.00)

^a^ (C) indicates central‐point replicates.

For film casting, 25 mL of the film‐forming solution was dispensed into silicone moulds (surface area: 7.5 cm^2^) using a glass pipette. The moulds were maintained at 25 °C for 6 h to allow trapped air bubbles to dissipate and then transferred to a forced‐air oven at 40 °C for 24 h. The dried films were carefully detached from the moulds and conditioned in a desiccator (50% relative humidity, 25 °C) until characterisation. The HPMC, CS and Gly concentrations were chosen based on the most frequent recommendations observed in the literature.^5^, ^7^, ^10^, ^19^, ^24^

### Factorial experimental design

A factorial design is widely used for efficient optimisation of film formulations because it reduces the number of experiments required and enables systematic exploration of component–response relationships.^40^ Accordingly, a 2^3^ factorial experimental design with four centre‐point replicates was employed to evaluate the effects of three independent variables, HPMC concentration (*X*₁), CS concentration (*X*₂) and Gly concentration (*X*₃), on two response variables: tensile strength and elongation at break. Each factor was tested at three coded levels: low (−1), centre (0) and high (+1), as detailed in Table 1. The design allowed assessment of both main effects and interaction effects among the variables. A total of 12 experimental runs were conducted in randomised order to minimise systematic error, and all formulations were prepared and tested under identical environmental conditions to ensure comparability.

### Statistical analysis

Data were analysed by analysis of variance (ANOVA; Statistica 8.0; StatSoft, 2008), with *α* = 0.05. When applicable, means were compared using Tukey's test (5%). In the 2^3^ factorial design, main and interaction effects were estimated and ranked to build Pareto charts and guide formulation selection.

### Mechanical tests of films

Stress (*σ*), strain (*ε*) and Young's modulus (*E*) were determined by tensile testing. Film thickness was measured using a digital micrometer (accuracy 0.001 mm), with three measurements taken at different points and the average value used in calculations.

Rectangular specimens (9 × 30 mm^2^) were tested at 25 °C using a universal testing machine (EMIC DL‐1000) according to ASTM D882‐02. The crosshead speed was set to 10 mm min^−1^, with a gauge length of 15 mm, and a load cell capacity of 0.5 kN.

Three specimens per formulation were tested, and average values were reported. Formulations exhibiting superior mechanical performance were selected for subsequent characterisation.

### Subjective analysis of films

Subjective analysis was performed through visual and tactile inspection, evaluating gloss, flexibility, tackiness, transparency and surface texture. Each attribute was scored on a four‐point scale, where 1 corresponded to the most favourable condition (e.g. very bright, very malleable) and 4 to the least favourable condition (e.g. not bright, not malleable). The criteria for each score are presented in Table 2. Three evaluators conducted the assessments independently, and any discrepancies were resolved by consensus after analysis by a fourth evaluator.

**Table 2:** Scoring system used in subjective analysis of monolayer polymeric blend films

Scale	Brightness	Malleability	Tackiness	Transparency	Smoothness/texture
1	Very bright	Very malleable	Very tacky	Very transparent	Smooth/velvety
2	Bright	Malleable	Tacky	Transparent	Smooth/soft
3	Slightly bright	Slightly malleable	Slightly tacky	Slightly transparent	Smooth/plasticised
4	Not bright	Not malleable	Not tacky	Not transparent	Rough

### Transparency test

A film specimen was cut to match the dimensions of a quartz cuvette and subdivided into strips (30 × 10 mm^2^). Each strip was fixed to the exterior of a pre‐cleaned, dried cuvette using transparent adhesive tape, ensuring full contact and no air bubbles. Optical measurements were taken at 560 nm using a UV–visible spectrophotometer (Even, model IL‐592).

For each formulation, three independent strips were measured (*n* = 3). A cuvette without any film was used as the control (blank). Transmittance at 560 nm was recorded and the mean value was reported for each sample. The adhesive tape used to secure the film strip was positioned outside the optical path, ensuring that it did not influence the transmittance measurements.

### Colorimetry

Colour was measured with a portable colourimeter (CR‐400, Konica Minolta, Japan). Four distinct positions along the length of each film specimen were assessed to capture spatial variation. Results are reported in the CIE Lab* colour space, where L* denotes lightness (0 = black, 100 = white), a* ranges from green (−) to red (+) and b* from blue (−) to yellow (+).

### Water solubility and absorption

Square film specimens (2 cm × 2 cm; area = 4 cm^2^) were prepared. Each specimen was pre‐dried in a forced‐air oven at 105 °C (±1 °C) for 1 h and cooled to room temperature in a desiccator before weighing on an analytical balance (*W*_i_; g). Specimens were placed in pre‐tared Petri dishes, covered with 60 mL of distilled water, and agitated on an orbital shaker (60 rpm) for 24 h. After immersion, specimens were removed, gently blotted on absorbent paper for 10 s without rubbing and weighed immediately (*W*_u_; g). They were then dried again in an oven at 105 °C to constant mass and re‐weighed (*W*_f_; g). Three independent specimens were tested per formulation (*n* = 3).

Water solubility was calculated as:

(1) $$\mathrm{WS}\;\left(\%\right)=\frac{\left(W_{i}-W_{f}\right)}{W_{i}}\times 100$$

where WS is the percentage of material dissolved in water, *W*_i_ is the initial dry weight and *W*_f_ is the final dry weight after immersion and re‐drying.

Water absorption (uptake) was calculated as:

(2) $$\mathrm{WA}\;\left(\%\right)=\frac{\left(W_{u}-W_{f}\right)}{W_{f}}\times 100$$

where WA is the percentage of water absorbed at the end of immersion, *W*_u_ is the wet weight after blotting and *W*_f_ is as defined above.

### Water vapour permeability (WVP)

WVP was determined according to ASTM E96/E96M (water method, wet‐cup).^41^ Circular film discs (25 mm in diameter) hermetically sealed the mouths of 50 mL polypropylene tubes (Falcon) containing 30 mL of distilled water; internal relative humidity (RH1) was 1.0. The exposed area *A* was the internal mouth area, measured with callipers. Assemblies were placed in a sealed desiccator at 25 ± 1 °C; external relative humidity was monitored with a digital hygrometer and maintained at 50 ± 2% (RH2 = 0.5). Each cup was weighed at *t* = 0 and *t* = 168 h, the mass change being Δ*W* = *m*_168_ *− m*_0_ (g). During the WVP analysis, all environmental conditions were maintained constant and mass loss was periodically monitored to verify and confirm linear WVP. The 168 h period was chosen to ensure that film WVP had no changes over long periods of time. Film thickness *E* (mm) was measured at multiple positions and averaged. The saturation vapour pressure of water at 25 °C was taken as *S* = 3.17 kPa, and the time interval as *T* = 168 h. Results are reported as mean ± standard deviation (SD) for *n* = 3 cups per formulation.

(3) $$\mathrm{WVP}=\frac{\Delta W\times E}{S\times T\times A\left(RH_{1}-RH_{2}\right)}\left[g\;\mathrm{mm}\;\mathrm{kP}a^{-1}\;h^{-1}\;m^{-2}\right]$$

### ATR‐FTIR spectroscopy

Attenuated total reflection Fourier transform infrared (ATR‐FTIR) spectra were collected with an IRTracer‐100 (Shimadzu) to examine functional groups at the film surface. Forty‐five scans were recorded over 600–4000 cm^−1^ at 8 cm^−1^ resolution using a small solid sample.

### Surface morphology

Surface morphology was examined using scanning electron microscopy (SEM; TM1000, Hitachi) operated at 15 kV. Films were cut into small pieces and mounted on pre‐cleaned circular metal stubs; each side was examined on separate mounts. A thin gold layer was deposited under vacuum (Q150R ES, Quorum), and the gold‐coated samples were subsequently imaged.

### Evaluation of film antimicrobial activity

Film discs (10 mm in diameter) were die‐cut from the films with a sterile punch and placed at the centre of Mueller–Hinton agar plates previously inoculated to obtain a uniform bacterial lawn of each test strain (Gram‐positive: *Staphylococcus aureus* ATCC 29213, *Enterococcus faecalis* ATCC 29212; Gram‐negative: *Escherichia coli* ATCC 10799, *Klebsiella pneumoniae* clinical isolate 153HU). Plates were incubated at 37 °C for 24 h. The growth‐inhibition zone diameter (mm) was measured including the 10 mm film disc. Positive controls (1% chlorhexidine solution and *Cinnamomum cassia* essential oil) were run in parallel (*n* = 3).

The selection of bacterial strains was intended to provide an initial and broad indication of the potential antimicrobial behaviour of the developed film. Standard Gram‐positive and Gram‐negative reference strains commonly used in studies on biobased antimicrobial films and active packaging were employed because they allow reproducible and comparative screening across materials with different compositions.

### Packaging setup and storage conditions for fresh‐cut mango

‘Tommy Atkins’ mangoes were harvested at physiological maturity from a commercial orchard in the São Francisco Valley (Juazeiro, Bahia, Brazil). This cultivar was selected because it is the most produced and exported in the region.^42^ After harvest, fruit were sanitised with 0.5 mL L^−1^ of a 10% available chlorine solution, then peeled, cut into cubes of approximately 2 cm edge length, gently mixed to ensure uniformity and washed again with 0.5 mL L^−1^ of a 10% available chlorine solution. Processed mangoes were surface‐dried at 25 °C.

Three packaging conditions were tested: (i) cubes placed in 100 mL food‐grade plastic cups without a sealing film (control); (ii) cubes in 100 mL plastic cups sealed with the test film (HPMC/CS+Gly; thickness of 62 ± 8 μm); and (iii) cubes in 100 mL plastic cups sealed with a commercial PVC film (thickness of 16 ± 2 μm). The experiment followed a completely randomised design with three packages (cups) per treatment, each package containing two fresh‐cut cubes. Samples were stored at 9 °C and evaluated at 0, 3, 6 and 9 days for headspace O₂, CO₂ and ethylene, as well as weight loss, colour, firmness, soluble solids and titratable acidity.

Headspace gases were measured directly in the package with a portable gas analyser (F‐960, Felix Instruments, USA). Empty cups and sealing films were individually pre‐weighed to determine tare mass. At each time point the gross mass of the closed package was recorded and the fruit mass was obtained by subtracting the corresponding tare; hence, weight loss was calculated on fruit mass only:

(4) $$\text{Weight loss}\;\left(\%\right)=\frac{\left(m_{0}-m_{t}\right)}{m_{0}}\times 100$$

where *m*_0_ is the tare‐corrected fruit mass at day 0 and *m*_*t*_ the tare‐corrected fruit mass at time *t*.

Pulp colour was measured with a portable colourimeter (CR‐400, Konica Minolta, USA) in the CIE L*a*b* colour space (CIE, 1976). Firmness was determined with a fruit hardness tester (PTR‐300 penetrometer, Instrutherm, Brazil) fitted with a 6 mm probe; values represent the force (N) required to penetrate the pulp to a 10 mm depth. Soluble solids were determined with a digital refractometer (PAL BX|ACID F5, Atago, Brazil) using 1 mL of strained juice and expressed as percentage (°Brix). Titratable acidity was measured with the same instrument using 1 mL of juice diluted 1:50 with distilled water; results were expressed as percentage. Data were analysed using ANOVA, and means were compared by Tukey's test (5%) where appropriate.

## RESULTS AND DISCUSSION

### Mechanical properties of HPMC/CS+Gly monolayer films

**Figure 1:**
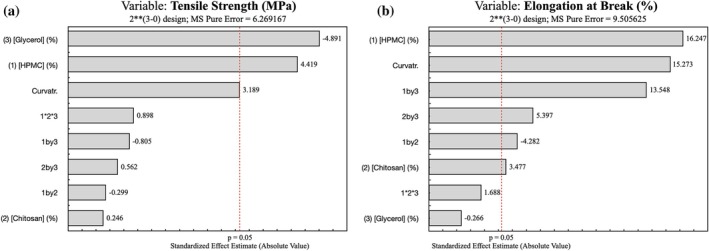
Pareto chart of TS (a) and EAB (b) of film formulations in response to different concentrations of HPMC, CS and Gly.

To evaluate how polymer and plasticiser levels affect mechanical behaviour, films were tested for tensile strength (TS) and elongation at break (EAB), and the formulation was selected by balancing stiffness (high TS) and extensibility (high EAB) (Table 3). A factorial analysis with a significance criterion of *P* < 0.05 was used to rank effects and to generate Pareto charts (Fig. 1).

**Table 3:** Tensile strength and elongation at break for 12 film formulations combining HPMC, CS and Gly

Film formulation (%HPMC/%CS/%Gly)	Tensile strength (MPa)	Elongation at break (%)
1.00/0.50/0.50	8.51 ± 0.19	72.37 ± 4.57
3.00/0.50/0.50	19.88 ± 0.45	91.27 ± 0.85
1.00/1.00/0.50	10.07 ± 0.90	81.20 ± 0.28
3.00/1.00/0.50	17.20 ± 0.82	74.07 ± 2.80
1.00/0.50/2.00	1.87 ± 0.10	34.17 ± 5.80
3.00/0.50/2.00	7.21 ± 0.85	104.78 ± 2.79
1.00/1.00/2.00	2.24 ± 0.42	59.17 ± 2.30
3.00/1.00/2.00	9.70 ± 0.44	118.47 ± 8.31
2.00/0.75/1.00 (centre point)	16.31 ± 0.82	105.29 ± 0.96
2.00/0.75/1.00 (centre point)	10.93 ± 0.01	106.00 ± 3.96
2.00/0.75/1.00 (centre point)	16.17 ± 2.89	111.47 ± 11.90
2.00/0.75/1.00 (centre point)	14.49 ± 0.48	110.33 ± 4.16

Across formulations, higher HPMC was associated with higher TS: the 3.00% HPMC films reached 19.88 ± 0.45 and 17.20 ± 0.82 MPa (samples 2 and 4), whereas lower‐HPMC counterparts showed lower TS (Table 3). Increasing Gly generally reduced TS and, in several cases, increased EAB; for example, 3.00/0.50/2.00 yielded 7.21 ± 0.85 MPa and 104.78 ± 2.79%, while 3.00/1.00/2.00 showed 9.70 ± 0.44 MPa and 118.47 ± 8.31%. These trends are consistent with reports that Gly, as a plasticiser, lowers intermolecular resistance in polysaccharide films and thereby reduces TS.^43^, ^45^

The centre‐point replicates (2.00/0.75/1.00) supported the overall pattern, with TS ranging from 10.93 to 16.31 MPa and EAB from 105% to 111%, indicating high extensibility around the design centre (Table 3).

Effect ranking from the factorial design is summarised in the Pareto charts. For TS, Gly and HPMC displayed the largest standardised effects, where Gly was negative and HPMC positive (Fig. 1(a)). For EAB, HPMC and CS contributed positively, particularly at higher levels (Fig. 1(b)).

A complementary subjective assessment (brightness, malleability, tackiness, transparency and surface texture) was used to aid screening; attributes were scored from 1 (high intensity/present) to 4 (low intensity/not present) (Table 4).

**Table 4:** Film brightness, malleability, tackiness, transparency and smoothness in response to different concentrations of HPMC, CS and Gly^a^

Sample (%HPMC/%CS/%Gly)	Brightness	Malleability	Tackiness	Transparency	Smoothness/texture
1.00/0.50/0.50	1	1	2	3	2
3.00/0.50/0.50	2	2	4	3	3
1.00/1.00/0.50	1	1	2	3	2
3.00/1.00/0.50	2	2	4	3	3
1.00/0.50/2.00	1	1	1	3	2
3.00/0.50/2.00	2	2	3	3	3
1.00/1.00/2.00	1	1	1	3	2
3.00/1.00/2.00	2	2	3	3	3
2.00/0.75/1.00 (centre point)	2	3	3	3	3
2.00/0.75/1.00 (centre point)	2	3	3	3	3
2.00/0.75/1.00 (centre point)	2	3	3	3	3
2.00/0.75/1.00 (centre point)	2	3	3	3	3

^a^ Each attribute was evaluated on a scale from 1 (high intensity/present) to 4 (low intensity/not present).

From this assessment, brightness varied between ‘very bright’ (score 1) and ‘bright’ (score 2) and was linked to HPMC level: 1% HPMC films were ‘very bright’, whereas 2–3% HPMC films were ‘bright’ (Table 4). Malleability scores increased from 1 to 2 to 3 as HPMC increased (that is, malleability decreased with higher HPMC). Tackiness rose with Gly and fell with more HPMC; notably, only samples 2 and 4 (both 3% HPMC, 0.5% Gly) were scored non‐tacky (4). Transparency scores were uniform (3) across formulations, and surface smoothness/texture was typically 2 to 3 (Table 4). These attributes matter for consumer acceptance and for optical systems in intelligent packaging.^46^, ^50^

Considering the mechanical outcomes together with the subjective evaluation, sample 2 (3% HPMC, 0.5% CS, 0.5% Gly) was selected for subsequent characterisation because it combined high TS with high EAB and exhibited favourable handling (non‐tacky, smooth surface).

### Physical and chemical characterisation of selected film

#### Film transparency and colour

The selected film, composed of HPMC at 3%, CS at 0.5% and Gly at 0.5% (hereafter HPMC/CS+Gly), appeared visually transparent with a faint yellow hue. In the CIE L*a*b* colour space, lightness was L* = 28.39 ± 0.25, indicating low lightness under the measurement conditions; the chromatic coordinates showed a slight shift towards green (a* = −0.24 ± 0.01) and towards yellow (b* = 0.55 ± 0.03).

Literature on chitosan‐based films reports an intrinsically yellow control (b* ≈ 10.35) and additive‐driven increases in b* and Δ*E* with reductions in L*.^35^ In CS/HPMC blend films containing sage leaf extract or nettle leaf extract, L* remains high overall but decreases with extract addition, while a* becomes more negative, b* increases and Δ*E* rises.^51^ Absolute L*a*b* values are method dependent owing to illuminant, observer, geometry, backing and thickness.

UV radiation promotes free‐radical formation and can degrade food constituents, affecting antioxidants, proteins, nutritional value, flavour and appearance.^52^ The film transmittance at 560 nm was 30.6 ± 1.04%, which indicates limited visible‐light transmission. While higher transparency may benefit consumer perception, reduced light transmission can help mitigate photo‐oxidative deterioration, as reported by Wu *et al*. and Wang *et al*.^53^, ^54^ Therefore, protecting packaged foods from UV exposure is important, and controlling visible‐light passage can be advantageous in practical applications, including active and intelligent packaging.^55^, ^56^

#### Water absorption and solubility of HPMC/CS+Gly film

Water absorption and water solubility are relevant attributes for films intended for fresh‐cut fruit applications, which typically require storage at high relative humidity to avoid dehydration.^5^ The HPMC/CS+Gly film showed water solubility of 38.74 ± 11.29% and water absorption of 420.83 ± 4.04% under the test conditions described in the section on Water solubility and absorption. These values reflect the hydrophilic character of the polysaccharide matrix.

Consistent with the literature, Liang *et al*. reported that films made exclusively from HPMC or CS tend to exhibit higher water solubility, whereas combining HPMC with CS markedly reduces solubility.^57^ This reduction has been attributed to increased intermolecular hydrogen bonding between amino groups in CS and hydroxyl groups in HPMC, which promotes a denser network that is less prone to dissolution.^58^, ^59^ In addition, compositional modifications with selected components, such as essential oils, have been reported to further decrease water solubility when the aim is to impart active and/or intelligent functionality to the material.^60^

#### Film WVP

Under wet‐cup conditions (25 °C; ΔRH = 0.5), approximately 4% of the initial 30 g of water per cup (≈1.2 g) permeated through the film over 7 days (0–168 h). Water barrier properties are crucial, especially in food packaging, to prevent excessive moisture loss from foods to the atmosphere.^61^ WVP has been widely studied due to its importance, and lower permeability values are generally recommended for high‐water‐content foods.^62^, ^65^

According to our results, the HPMC/CS+Gly film showed a WVP of 1.045 ± 0.064 g mm kPa^−1^ h^−1^ m^−2^ (*n* = 3). This magnitude is consistent with the known hydrophilicity of polysaccharide films: the polymer matrix contains abundant hydroxyl and amino groups that interact with water molecules, facilitating vapour transport through the film structure.^66^

However, films combining HPMC and CS with 7.5% sage leaf extract or 7.5% nettle have reported to have lower WVP values (0.266 and 0.408 g mm kPa^−1^ h^−1^ m^−2^, respectively).^34^ Although formulations and testing conditions may differ across studies, these results indicate that incorporating selected natural compounds can reduce WVP and may enhance performance for fresh‐cut fruit packaging.

#### ATR‐FTIR spectroscopy

**Figure 2:**
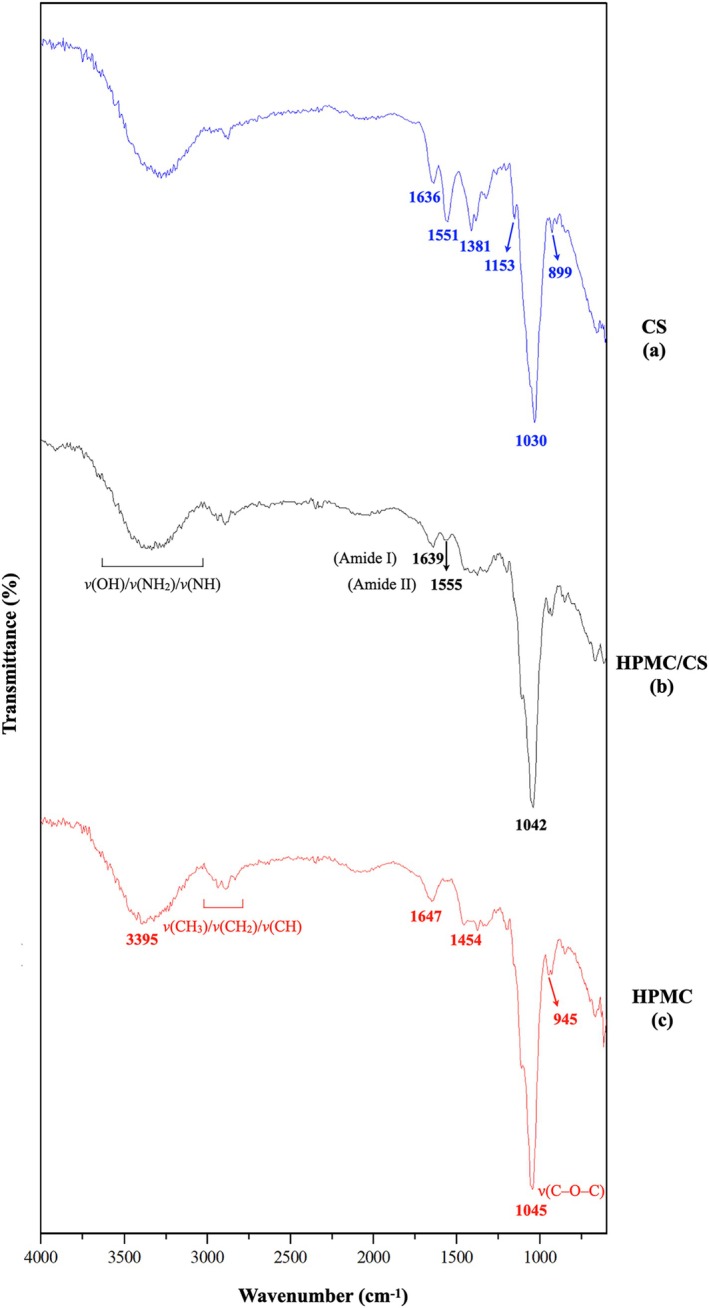
ATR‐FTIR spectra of films based on (a) CS with Gly (CS), (b) HPMC/CS with Gly (HPMC/CS) and (c) HPMC with Gly (HPMC).

ATR‐FTIR spectra were collected as described in the section on ATR‐FTIR spectroscopy to characterise functional groups in the films (Fig. 2). Three specimens were analysed: the selected HPMC/CS+Gly film (3% HPMC, 0.5% CS, 0.5% Gly) and two reference films prepared for band assignment: HPMC+Gly (1.75% HPMC, 0.5% Gly) and CS+Gly (1.75% CS, 0.5% Gly).

For the CS film (Fig. 2(a)), distinct bands at 1636 and 1551 cm^−1^ are assigned to amide I (*ν*(C=O)) and amide II (*δ*(N—H) + *ν*(C—N)), respectively. The band at 1381 cm^−1^ can be attributed to *ν*(C—O) of primary alcohols (—CH₂OH) and to symmetric bending of residual CH₃ groups. Features between 1153 and 899 cm^−1^ are consistent with saccharide structures and *β*‐glycosidic linkages of CS.^34^, ^67^, ^68^

For the HPMC film (Fig. 2(c)), a strong band at 1045 cm^−1^ corresponds to *ν*(C—O—C) of the pyranose ring.^34^, ^69^ The signal near 945 cm^−1^ is associated with ether‐related vibrations and appears as a weak feature overlapping the 1045 cm^−1^ band. A broad band at *ca* 3395 cm^−1^ arises from *ν*(OH) of hydrogen‐bonded chains.^27^, ^34^ Bands in the 3000–2800 cm^−1^ region are attributed to *ν*(CH₃)/*ν*(CH₂)/*ν*(CH). The band at 1647 cm^−1^ is commonly associated with bound water,^34^, ^70^ and that at 1454 cm^−1^ with *δ*(CH₃), close to *δ*(CH₂).

The HPMC/CS blend spectrum (Fig. 2(b)) differs from those of the individual films by slight band shifts and intensity changes. Relative to CS, amide I shifts from 1636 to 1639 cm^−1^ and amide II from 1551 to 1555 cm^−1^; the broad *ν*(OH)/*ν*(NH)/*ν*(NH₂) envelope also changes. Relative to HPMC, the strong carbohydrate band appears at *ca* 1042–1045 cm^−1^. These shifts and broadenings indicate altered hydrogen‐bonding environments consistent with intermolecular interactions between CS and HPMC, supporting miscibility/compatibility in the blend.^34^, ^57^

### Surface morphology

**Figure 3:**
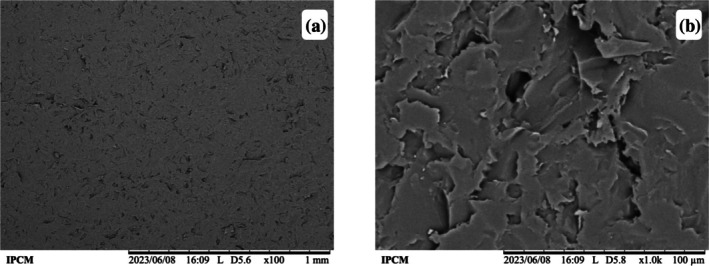
Surface SEM images of HPMC/CS+Gly film. Images were taken at ×100 (a) and ×1000 (b).

The surface morphology of the selected HPMC/CS+Gly monolayer film was examined using SEM (Fig. 3). SEM micrographs are commonly used to assess blend compatibility and possible phase separation in polymeric films, since interfacial discontinuities and microdomains may appear as surface irregularities that can influence mechanical behaviour.^71^

At ×100 (Fig. 3(a)), the surface appears continuous, with fine, irregular relief distributed across the field. At ×1000 (Fig. 3(b)), a more pronounced roughness is evident, with fragmented, flake‐like features and local micro‐irregularities; distinct second‐phase domains are not apparent at these magnifications. Similar surface features have been reported for CS/HPMC systems.^72^, ^73^ In contrast, films composed solely of CS or solely of HPMC are generally described as smoother and more homogeneous under comparable imaging conditions.^63^, ^71^

### Assessment of antimicrobial activity through film contact

Antimicrobial activity was evaluated by the disc diffusion method (Evaluation of film antimicrobial activity section) using *Escherichia coli* (ATCC 10799), *Klebsiella pneumoniae* (clinical isolate 153HU), *Staphylococcus aureus* (ATCC 29213) and *Enterococcus faecalis* (ATCC 29212). Positive controls were 1% chlorhexidine and *Cinnamomum cassia* essential oil.^74^, ^75^, ^76^ Inhibition was assessed from the diameter of the growth‐inhibition zone (mm), measured including the 10 mm film disc. By our *a priori* criterion, haloes of <15 mm were classified as non‐inhibitory (Table 5). These strains were chosen to provide an initial indication of broad‐spectrum antimicrobial behaviour, and to allow comparative assessment using reference organisms widely employed in studies of biobased antimicrobial films. Although these organisms do not represent the full spoilage microbiota of mango, they enable a consistent laboratory screening of the film.

**Table 5:** Antibacterial activity of HPMC/CS+Gly film determined according to the disc diffusion method

Sample	Diameter of growth inhibition halo (mm)			
	*Escherichia coli*	*Klebsiella pneumoniae*	*Staphylococcus aureus*	*Enterococcus faecalis*
HPMC/CS+Gly	—	11^a^	—	—
*Cinnamomum cassia*	20	33	26	30
Chlorhexidine (1%)	20	25	21	21

a Halos with a diameter < 15 mm should not be considered as indicating inhibitory activity.

The diameter of the growth‐inhibition halo was measured in addition to the 10 mm diameter of the HPMC/CS+Gly film disc. *Cinnamomum cassia* essential oil and chlorhexidine were used as positive controls against *E. coli*, *K. pneumoniae*, *S. aureus* and *E. faecalis*.

The HPMC/CS+Gly film (3% HPMC, 0.5% CS, 0.5% Gly) did not inhibit *E. coli*, *S. aureus* or *E. faecalis*, and produced an 11 mm clearing against *K. pneumoniae*, which is below the 15 mm cut‐off and therefore non‐inhibitory. In contrast, the positive controls yielded clear haloes: *C. cassia* essential oil of 20–33 mm depending on the strain, and 1% chlorhexidine of 20–25 mm (Table 5).

These findings align with reports that HPMC lacks intrinsic antibacterial activity,^73^, ^77^ while CS can be antimicrobial in a manner dependent on degree of deacetylation, molecular weight and environmental conditions such as pH, ionic strength and reactive solutes.^66^, ^78^, ^79^, ^80^, ^81^, ^82^ Under the present composition (0.5% CS) and test conditions, measurable inhibition was not observed. To enhance antibacterial performance in HPMC/CS films, incorporation of established active agents such as essential oils may be considered, either alone or in combination with metal oxide nanoparticles; such strategies have been reported to strengthen antimicrobial effects in polysaccharide‐based films.^83^, ^84^ Targeted studies will be required to optimise composition and to elucidate mechanisms when CS is combined with HPMC and Gly in food‐packaging films.^83^, ^84^

### Moisture‐management performance in fresh‐cut mango

**Figure 4:**
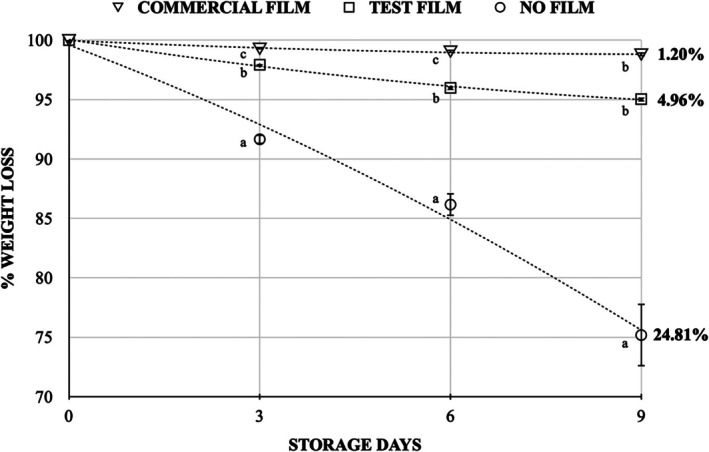
Weight loss of fresh‐cut mango during 9 days of storage at 9 °C. Fresh‐cut samples were stored in 100 mL food‐grade plastic cups without a sealing film (control), sealed with HPMC/CS+Gly (test film) or sealed with commercial PVC film. Values are means ± SD (*n* = 3 packages per treatment).

Fresh‐cut mango cubes were stored at 9 °C for 9 days under three conditions: the HPMC/CS+Gly test film (3% HPMC, 0.5% CS, 0.5% Gly), a commercial PVC film or no film (control). Weight loss increased over time in all treatments, but packaging markedly changed the magnitude of loss (Table 6; Fig. 4). At day 3 the values were 8.34% for no film, 2.07% for the test film and 0.72% for PVC. On day 6 they were 13.84%, 4.01% and 0.96%, respectively. On day 9 they were 24.81%, 4.96% and 1.20%, respectively. Thus, the HPMC/CS+Gly film reduced dehydration by approximately fivefold relative to the unpackaged control, while PVC gave the lowest losses throughout. This pattern is consistent with the typically higher WVP of polysaccharide films compared with PVC.^85^ Limiting water loss is technologically relevant because mass losses of 5–10% commonly render produce unmarketable and accelerate quality deterioration, including browning, textural softening and flavour loss.^86^, ^88^

**Table 6:** Physicochemical quality of fresh‐cut mango during 9 days of storage at 9 °C

Condition	Weight loss (%)	*L*	*a*	*b*	Firmness (N)	Soluble solids (%)	Acidity (%)	O_2_ (%)	CO_2_ (%)	Ethylene (ppm)
*At 0 day*										
Initial untreated	0.00	78.55	−5.29	69.62	21.55	11.33	0.49	21.00	0.03	0.00
*After 3 days*										
No film	8.34 ^a^	75.32 ^a^	−2.88 ^a^	71.70 ^a^	20.40 ^a^	13.00 ^a^	0.52 ^ab^	21.00 ^a^	0.03 ^b^	0.00 ^a^
Test film	2.07 ^b^	73.19 ^a^	−1.10 ^a^	75.24 ^a^	18.78 ^a^	14.20 ^a^	0.56 ^a^	20.96 ^a^	0.23 ^b^	6.16 ^a^
Commercial film	0.72 ^c^	77.35 ^a^	−3.71 ^a^	69.78 ^a^	14.77 ^a^	13.10 ^a^	0.49 ^b^	14.66 ^b^	3.20 ^a^	31.95 ^a^
CV (%)	6.40	3.55	52.68	2.88	24.75	4.38	4.31	9.68	32.00	154.95
*After 6 days*										
No film	13.84 ^a^	77.26 ^a^	−3.72 ^a^	64.07 ^a^	20.21 ^a^	13.70 ^a^	0.50 ^a^	21.00 ^a^	0.03 ^b^	0.00 ^b^
Test film	4.01 ^b^	76.43 ^a^	−3.26 ^a^	64.09 ^a^	20.14 ^a^	13.34 ^a^	0.49 ^a^	20.43 ^a^	0.36 ^b^	1.20 ^ab^
Commercial film	0.96 ^c^	77.34 ^a^	−3.39 ^a^	67.03 ^a^	15.18 ^a^	12.44 ^a^	0.43 ^a^	12.70 ^a^	1.97 ^a^	2.26 ^a^
CV (%)	8.87	5.57	44.22	11.75	58.55	7.40	21.53	21.15	71.36	50.29
*After 9 days*										
No film	24.81 ^a^	69.39 ^a^	−0.79 ^a^	65.28 ^a^	15.26 ^a^	15.96 ^a^	0.54 ^a^	21.00 ^a^	0.03 ^a^	0.00 ^c^
Test film	4.96 ^b^	78.13 ^a^	−2.49 ^a^	58.51 ^a^	19.95 ^a^	13.84 ^b^	0.52 ^a^	19.60 ^a^	0.74 ^a^	2.50 ^b^
Commercial film	1.20 ^b^	73.33 ^a^	−0.81 ^a^	59.51 ^a^	20.17 ^a^	12.43 ^b^	0.42 ^a^	17.30 ^a^	1.64 ^a^	3.56 ^a^
CV (%)	14.67	7.16	161.45	9.69	55.98	4.32	11.74	15.89	118.65	20.65

Means followed by the same superscript letter on each day of storage are statistically equal according to Tukey's test (5%).

Fresh‐cut samples were stored in 100 mL food‐grade plastic cups without a sealing film (control), sealed with HPMC/CS+Gly (test film) or sealed with commercial PVC film.

Packaging did not affect colour (L*, a*, b*) or firmness within each storage day, as indicated by the same superscript letters for treatments in Table 6 (ANOVA and Tukey, 5%). These attributes are recognised indicators of freshness and strongly influence consumer acceptance in fruit products.^89^, ^90^ For soluble solids, no differences were detected up to day 6. At day 9, values were higher without film (15.96%) than with the test (13.84%) or PVC (12.43%) films (*P* < 0.05). This pattern is consistent with concentration effects from the greater water loss in the unpackaged fruit. In mango, the rise in soluble solids reflects starch hydrolysis to sugars during ripening.^91^ Titratable acidity showed a transient difference at day 3 with that for PVC slightly lower than that for the test film, with no consistent treatment effect thereafter (Table 6).

Headspace measurements indicate limited atmosphere modification, particularly for the HPMC/CS+Gly film. O₂ remained close to ambient, approximately 21%, and CO₂ was at or below 0.74% by day 9. The PVC film transiently lowered O₂ to 14.66% at day 3 and increased CO₂ to 3.20% at day 3, trending back towards ambient by day 9. Ethylene accumulated in sealed packages and was numerically higher under PVC than under the test film at day 9 (3.56 *versus* 2.50 ppm), while the open control remained at 0 ppm (Table 6). The small changes in O₂ and CO₂, particularly for the HPMC/CS+Gly film, explain the lack of ripening delay, with colour and firmness unchanged, and are consistent with reports that films with low resistance to O₂ and CO₂ diffusion have limited impact on respiration and ripening.^92^, ^95^ Low levels of O₂ and high levels of CO₂ in the storage atmosphere are known to inhibit fruit respiration and ethylene synthesis, which results in slow ripening changes during storage and shelf life.^19^, ^96^, ^97^ These results suggest that the performance of the HPMC/CS+Gly film in maintaining fruit quality traits, such as colour and firmness, could be enhanced by increasing its resistance to O₂ and CO₂ diffusion, as well as by reducing its resistance to ethylene or enabling ethylene absorption within the package headspace,^19^, ^24^ which will be the focus of future studies.

Overall, the HPMC/CS+Gly film provided effective water‐barrier performance relative to no film, although it was inferior to PVC, and it exerted minimal gas‐barrier effects. For applications that aim to curb dehydration and, where relevant, to modulate the internal atmosphere to slow ripening, future work should tailor the film composition to increase resistance to O₂ and CO₂ diffusion, thereby achieving a headspace with lower O₂ and higher CO₂ that more effectively suppresses respiration.^92^, ^95^

## CONCLUSIONS

This study demonstrates the feasibility of a biodegradable HPMC/CS film plasticised with Gly for fresh‐cut mango. The selected monolayer formulation showed polymer–polymer compatibility by ATR‐FTIR, high optical clarity, reduced water solubility and WVP suited to moisture control. Used as a sealing lid at 9 °C for 9 days, it reduced weight loss by about fivefold compared with unpackaged fruit and maintained fruit colour and firmness during storage, while approaching the dehydration‐limiting performance of commercial PVC. Overall, the HPMC/CS+Gly film is a credible moisture‐management solution for fresh‐cut mango and a solid platform for future optimisation aimed at strengthening gas‐barrier properties and, where appropriate, adding active functionality. Future studies should aim to enhance the resistance of HPMC/CS+Gly films to O₂ and CO₂ diffusion, while reducing the resistance to ethylene or enabling ethylene absorption within the package headspace. These improvements are expected to increase the film's effectiveness in delaying tissue ripening and quality deterioration, thereby reducing postharvest losses and extending the time available for storage, transport, marketing and consumption. Although regulatory compliance for food contact and migration was not assessed, the polymers used (HPMC and CS) are widely recognised as safe for food applications, and migration testing may be considered in future work.

## FUNDING INFORMATION

This study was financed in part by the Coordenação de Aperfeiçoamento de Pessoal de Nível Superior – Brasil (CAPES) – Finance Code 001.

## CONFLICT OF INTEREST

The authors assert the absence of known conflicting financial interests or personal relationships that might have been perceived to impact the work presented in this paper.

## AUTHOR CONTRIBUTIONS

AGP: conceptualisation, data curation, formal analysis, investigation, methodology, validation, writing – original draft. GVLJ: data curation, investigation. ACS: data curation, investigation. FSF: data curation, investigation. PVMR: data curation, investigation. MPC: formal analysis, software, writing – original draft. STF: data curation, formal analysis, investigation, validation, writing – original draft. DFMN: conceptualisation, data curation, formal analysis, investigation, methodology, project administration, resources, software, supervision, validation, writing – original draft, writing – review & editing.

## Data Availability

The data that support the findings of this study are available from the corresponding author upon reasonable request.
